# Complete Genome Sequence of the Pokeweed Mosaic Virus (PkMV)-New Jersey Isolate and Its Comparison to PkMV-MD and PkMV-PA

**DOI:** 10.1128/genomeA.00929-16

**Published:** 2016-09-08

**Authors:** Rong Di

**Affiliations:** Department of Plant Biology and Pathology, Rutgers, The State University of New Jersey, New Brunswick, New Jersey, USA

## Abstract

Pokeweed mosaic virus (PkMV) causes systemically mosaic symptoms on pokeweed (*Phytolacca americana* L.) plants. The genome of the PkMV-NJ (New Jersey) isolate was cloned by PCR and sequenced by the Sanger sequencing method. The sequence comparison indicates that PkMV-NJ is more divergent from the other two sequenced isolates, PkMV-MD and PkMV-PA.

## GENOME ANNOUNCEMENT

Pokeweed mosaic virus (PkMV), first described in 1969 (1), infects the pokeweed plant (*Phytolacca americana* L.) causing mosaic symptoms (1). PkMV was identified as flexuous particles about 776 nm in length (2). Electron microscopy of infected pokeweed leaf cells showed that PkMV caused cytoplasmic inclusion bodies (3). These data indicate that PkMV is a member of the *Potyvirus* in the family *Potyviridae* (4). The genome sequences of PkMV-MD and PkMV-PA isolates have recently been completed, confirming PkMV as a potyvirus (5). Several pokeweed antiviral proteins including PAP, PAPII, PAPIII, and PAP-S have been isolated from pokeweed, and the antiviral activity of PAP has been extensively studied (6). It is known that PAP is synthesized in the cytoplasm and extruded into the cell wall matrix (7). Up to date, PkMV seems to be the only virus reported to cause pokeweed mosaic disease. It is interesting to study how PkMV, reportedly transmitted by aphids (2), evades PAP antiviral activity and causes systemic mosaic symptoms in pokeweed plants. To this end, the New Jersey isolate of PkMV, PkMV-NJ, was purified, its viral genome was cloned, sequenced, and compared to PkMV-MD and PkMV-PA.

PkMV-NJ was purified from infected pokeweed leaves collected from the Rutgers Gardens in New Jersey. Briefly, the leaves were pressed with a juicer in borate buffer containing 0.5 M boric acid (pH 8.0), 0.15% thioglycolic acid, and 0.01 M sodium diethylthiocarbonate. After stirring in 0.5 vol of chloroform, PkMV-NJ was purified from the juice by PEG (MW 6000) and NaCl precipitation and alternate low-speed (6000 × *g*, 10 min) and high-speed (8000 × *g*, 20 min) centrifugations. The PkMV-NJ viral particles were further purified and collected with an Amicon 100K MWCO centrifugal filter. Viral RNA was isolated with the Trizol Reagent (Ambion), and used to produce first strand cDNA with the High Capacity cDNA synthesis kit (Applied Biosystems) and the oligo d(T) primer. After comparing the sequences of PkMV-MD and PkMV-PA, five sets of PCR primers were designed based on their most-conserved sequences to amplify the PkMV-NJ genome in five fragments. The PCR amplicons were cloned into pGEMT-easy (Promega) and sequenced by the Sanger sequencing method (Genewiz). New primers were designed within the amplicons to re-clone and sequence the fragments flanked by the first five sets of primers. The complete PkMV-NJ cDNA sequence was obtained and aligned to the sequences of PkMV-MD (JQ609096) and PkMV-PA (JQ609095) (5) by NCBI BLAST. It has been shown that PkMV-MD and PkMV-PA differ by 121 nucleotides (nt) and 12 amino acids (5). However, the analysis shows that out of the 9,512 nt, PkMV-NJ differs by 237 nt and 314 nt from PkMV-MD and PkMV-PA, respectively. PkMV-NJ also differs by 46 and 55 amino acid residues in the 3,056-residue polyprotein from PkMV-MD and PkMV-PA, respectively. Most of the sequence divergence of PkMV-NJ from PkMV-MD and PkMV-PA are at the 5′ end of the genome.

### Accession number(s).

The complete genome sequence of PkMV-NJ has been deposited in GenBank under the accession no. KU133475. The version described in this paper is the first version.
